# Practitioner perceptions of biodiversity criteria for solar suitability analyses in the United States

**DOI:** 10.1038/s44185-026-00133-w

**Published:** 2026-05-24

**Authors:** Daphne Condon, Michael O. Levin, Adam B. Smith, Toni Lyn Morelli, Noah Z. Krasner, Emma Forester, Chevon Holmes, Benjamin P. Narwold, Elizabeth L. Kalies, Grace C. Wu, Meaghan R. Gade, Roland Kays, Freya Robinson, Rebecca R. Hernandez

**Affiliations:** 1 Global Ecology and Sustainability Lab, Land, Air & Water Resources Department, University of California, Davis, CA USA; 2 Wild Energy Center, University of California, Davis, CA USA; 3 Department of Ecology, Evolution, and Environmental Biology, Columbia University, New York, NY USA; 4 Center for Conservation and Sustainable Development, Missouri Botanical Garden, St. Louis, MO USA; 5 U.S. Geological Survey, Northeast Climate Adaptation Science Center, Amherst, MA USA; 6 The Nature Conservancy, Durham, NC USA; 7 Environmental Studies, University of California Santa Barbara, Santa Barbara, CA USA; 8 Association of Fish and Wildlife Agencies, Washington, DC USA; 9 North Carolina Museum of Natural Sciences, Raleigh, NC USA; 10 Department of Forestry and Environmental Resources, NC State University, Raleigh, NC USA

**Keywords:** Climate sciences, Ecology, Ecology, Environmental sciences, Environmental social sciences

## Abstract

Acceleration of large-scale solar energy deployment can pose competition for land with biodiversity conservation areas. Solar suitability analyses (SSAs) help identify low-conflict zones for solar development, yet limited work defines which biodiversity-relevant criteria (BRCs) are essential for SSAs or whether supporting data are available. We convened a United States-based Delphi panel of practitioners with expertise in biodiversity and renewable energy to identify BRCs that are essential across SSAs (‘core’) and data- or scale-limited (‘peripheral’). Practitioners identified 16 core and 13 peripheral BRCs. Core criteria primarily aligned with regulatory frameworks, while peripheral BRCs reflected context-dependent ecological attributes lacking consistent and scalable data. Open-access data were available for 14 core criteria across 10 databases. Our assessment of US-based SSAs revealed that 10 included core BRCs. Our findings indicate a need for improved access to fine-scale biodiversity data and coordination with agencies to improve SSAs.

## Introduction

Solar energy is in an era of tremendous growth. Global installed photovoltaic solar capacity has expanded sixteen-fold in the last decade, contributing nearly 7% of electricity generation in 2024^1^. In the United States (US), solar deployment is primarily led by large-scale photovoltaic solar energy production (i.e., >1-MW capacity. Herein, “LSS”)^2,3^. Scenarios depicting full decarbonization of the US anticipate photovoltaic solar deployment to exceed 1500 GW by 2050, representing a near 1300% increase from the installed capacity in 2024^4,5^. Development of LSS has supported valuable progress on the United Nations Sustainable Development Goals (SDGs) by improving clean energy access (SDG 7) and mitigating impacts of climate-harming emissions (SDG 13).

The expansion of LSS introduces complex land-use and land cover-change-related conflicts with biodiversity conservation. Land conversion for human use is considered the leading driver of global biodiversity loss^6–9^, with over 80% of terrestrial ecosystems already transformed^10^. Landscapes well-suited for large-scale solar (e.g., inexpensive, flat, highly irradiated, and with access to existing transmission infrastructure)^11^ may overlap with those necessary to support biodiversity. In such cases, LSS can fragment habitats, disrupt ecological processes, and exacerbate species declines^8,12–14^. Many prospective LSS projects face local opposition regarding real and perceived ecological risks^15,16^, which may delay or halt development entirely^17,18^. In the United States, national conservation targets, such as the “30-by-30” initiative to conserve 30% of lands and waters by 2030, further elevate the importance of balancing renewable energy development with biodiversity protection. Although this target has since been rescinded, states continue to push progress toward these aims^19^. Addressing land-use and land-cover change-related conflicts between large-scale solar and conservation spaces remains a key consideration for governments striving for equitable clean energy deployment that does not undermine parallel SDGs related to biological conservation (e.g., Life on Land, SDG 15).

**Fig. 1:**
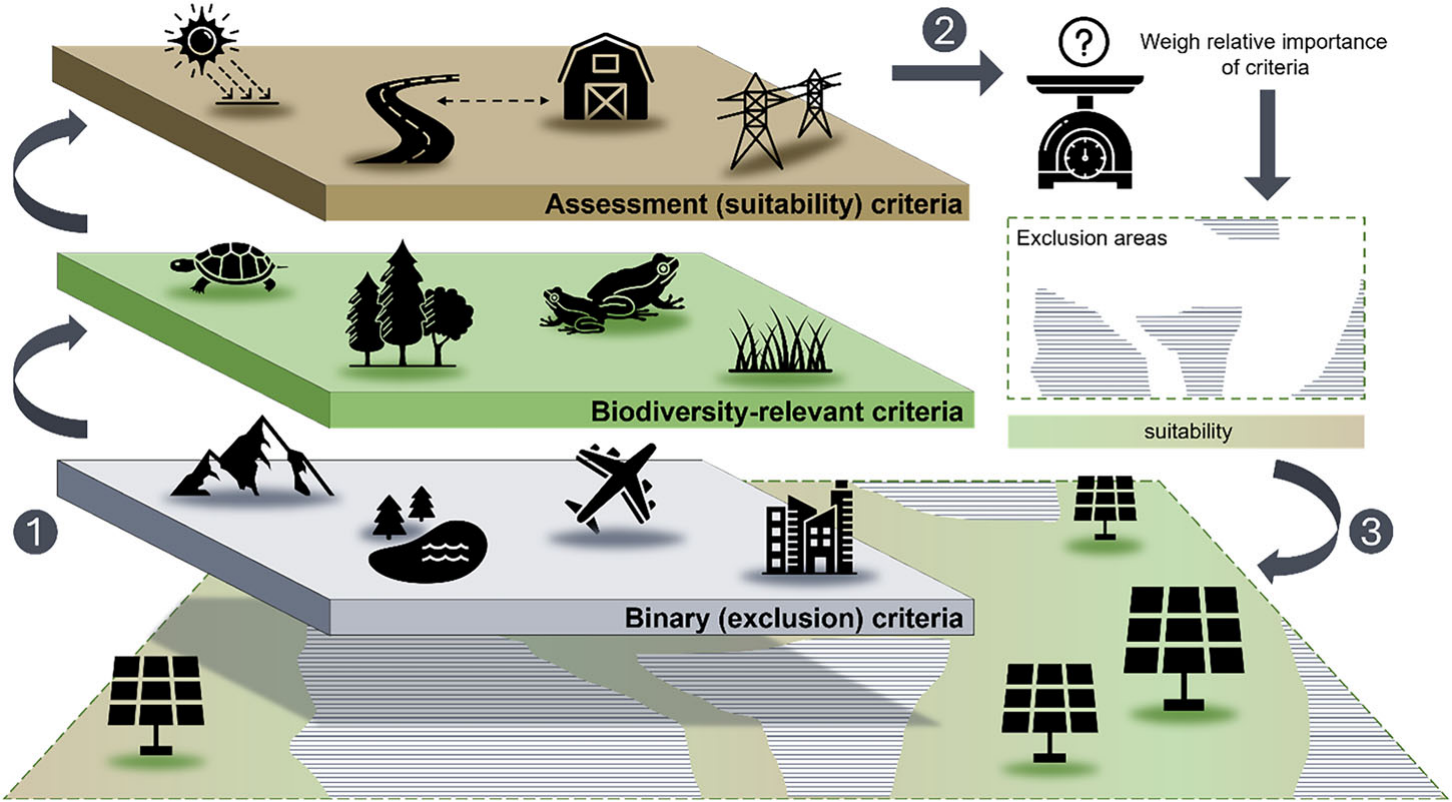
Example procedure flow for a solar suitability analysis. The process begins with selecting binary and assessment criteria, including biodiversity-relevant factors such as slope, restricted land uses, taxa of interest, critical species habitat, irradiance, and proximity to notable land uses. Criteria are then weighed to generate spatially explicit suitability and exclusion layers, which are combined to identify potential sites for large-scale solar facilities. Icons from NounProject.com™.

A common approach to addressing these conflicts is the solar suitability analysis (SSA), a spatial assessment that evaluates social, environmental, technical, and economic inputs to identify optimal siting zones^20^. These analyses use binary exclusionary criteria (e.g., legally protected areas, unsuitable slopes) and continuous assessment criteria (e.g., irradiance, land cover, proximity to infrastructure) to weigh trade-offs among competing land uses (Fig. 1)^21,22^. In practice, SSAs can offer decision support for early-stage site screening for large-scale solar projects by identifying regions of low developmental risk^23^. Depending on the application, SSAs may be conducted proactively to identify suitable regions at a landscape scale or applied in the context of individual project proposals. Governments or research institutions may conduct these assessments to inform landscape-scale planning guidance, while private developers or consultants may execute solar suitability analyses to support project-specific site screening. Findings from early-stage site screening SSAs can be incorporated within formal siting or zoning frameworks, such as the designation of inclusionary Solar Energy Zones, or provide guidance to local governments and developers to avoid siting LSS where projects may instigate adverse landscape impacts^24,25^. Importantly, solar suitability analyses typically serve as advisory tools, whereas the formal approval of LSS projects rests with permitting entities and is subject to federal, state, and local regulations.

Increasingly, solar suitability analyses are addressing the interactions between biological conservation and LSS development by incorporating environmental and ecological factors needed to maintain or restore biodiversity (herein, biodiversity-relevant criteria, or “BRCs”). These factors may include types of land under legal protection from developmental pressures (e.g., protected areas), habitat identified as important for wildlife (e.g., areas of critical environmental concern), and biodiversity metrics (e.g., presence of taxa of interest), among others (see Supplementary Item 1 for a full list). The evaluation of BRCs is typically conducted at the LSS project level to ensure regulatory compliance (e.g., the presence of threatened and endangered species’ critical habitat may trigger the federal Endangered Species Act). However, BRCs may also be integrated within solar suitability analyses targeted at informing landscape-scale planning guidance to identify areas with potential ecological conflicts earlier in the planning process and shift development away from areas of high biodiversity value. Despite this, BRCs remain inconsistently applied in solar suitability analyses. Across over 150 peer-reviewed studies conducting SSAs (herein, “SSA-related studies”), biodiversity-relevant criteria only accounted for roughly 4 and 29% of assessment and exclusionary criteria, respectively^20^. Despite these studies offering valuable SSA guidance, variability in biodiversity-relevant criteria hampers the comparability and ecological relevance of solar suitability analysis outcomes.

Given the projected scale of large-scale solar expansion and the uneven use of BRCs in solar suitability analyses, there is a clear need to establish a more consistent baseline for BRC inclusion in SSAs. The purpose of this study is to identify BRCs that are essential to every solar suitability analysis (herein, “core criteria”) and those that are limited by data or site-specificity (herein, “peripheral criteria”) in the US, along with associated datasets and relevant federal legislation. Focusing on the US enables us to examine large-scale solar siting within a single federal regulatory framework while addressing a national context where rapid LSS expansion and significant biodiversity threats converge^26,27^. We conduct a Delphi assessment, a structured, iterative expert elicitation process used to identify consensus on topics among participants^28^. By synthesizing input from 15 practitioners across academic, governmental, and NGO backgrounds, we examine (1) which criteria practitioners most consistently prioritize for SSAs and (2) the datasets and legislative frameworks that support Biodiversity-Relevant Criterion application in the US context. Further, we compare practitioner-identified criteria with those used in SSA-related studies. Our findings offer a practical baseline for aligning LSS siting decisions with biodiversity priorities as renewable energy buildout accelerates.

## Results

### Delphi-style focus group

Fifteen practitioners participated in our Zoom-based focus group on August 4th, 2023. Of these fifteen, only 10 contributed to the final Delphi round held after the focus group. Some of this dropout may be attributed to participation fatigue owing to the four complex analysis rounds.

**Fig. 2:**
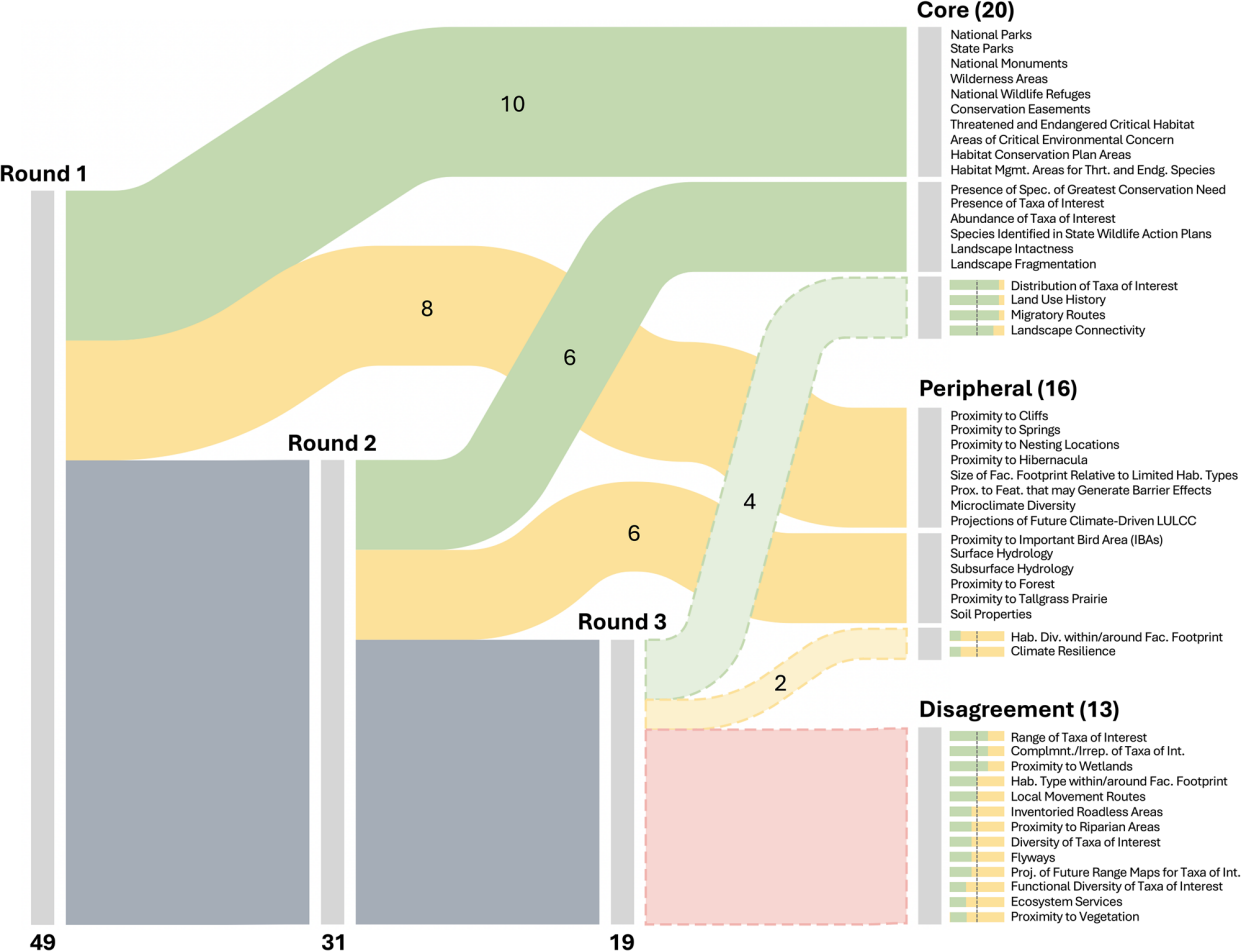
Criteria agreement flow by the Delphi round. Numbers within the flows indicate the criteria allocated to each section per round. All criteria that did not achieve 100% consensus are shown with a bar representing the votes toward either core (green) or peripheral (orange). Those with 80–90% consensus are grouped with their majority vote. Definitions are provided for all biodiversity-relevant criteria in Supplementary Item 1.

The first Delphi discussion round produced full consensus on 10 core criteria and 8 peripheral criteria (Fig. 2). Within the core criteria group, practitioners identified biodiversity-relevant criteria related to federal and state legal compliance; examples include national and state parks, wilderness areas, and conservation easements. Conversely, practitioners allocated a mix of BRCs with limited data and site-specific attributes into the peripheral criteria group, including proximity to hibernacula, microclimate diversity, and projections of future climate-driven land use and land cover change. In total, 31 disputed BRCs moved on to the following round.

In the second Delphi discussion round, practitioners reached a full consensus on six core and six peripheral criteria, the highest consensus rate relative to the total criteria discussed among all Delphi rounds (37%). Core criteria in this round exhibited weaker or indirect connections to federal regulations; instead, these criteria linked to non-binding and subnational mandates, like State Wildlife Action Plans or agency guidance. Peripheral criteria tended to reflect general ecological principles or emerging conservation science. In total, 19 disputed criteria continued to the third round.

The third Delphi round did not produce full consensus on any criteria, indicating a stopping point. Four core and two peripheral criteria reached near-consensus (80 or 90%) in this round. Of these criteria, land use history, landscape connectivity, migratory routes, and distribution of taxa of interest leaned toward a core classification. Like the core criteria from the second round, these criteria exhibit a closer linkage to non-binding and subnational mandates. Alternatively, climate resilience and habitat diversity within and around the facility footprint were considered peripheral by the plurality. These criteria tend toward site-specificity and may generate methodological ambiguity at screening scales. Thirteen criteria still faced classification disagreement, with the greatest split (50/50) exhibited by local movement routes and habitat type within and around the facility footprint.

### Data repository collection

**Fig. 3:**
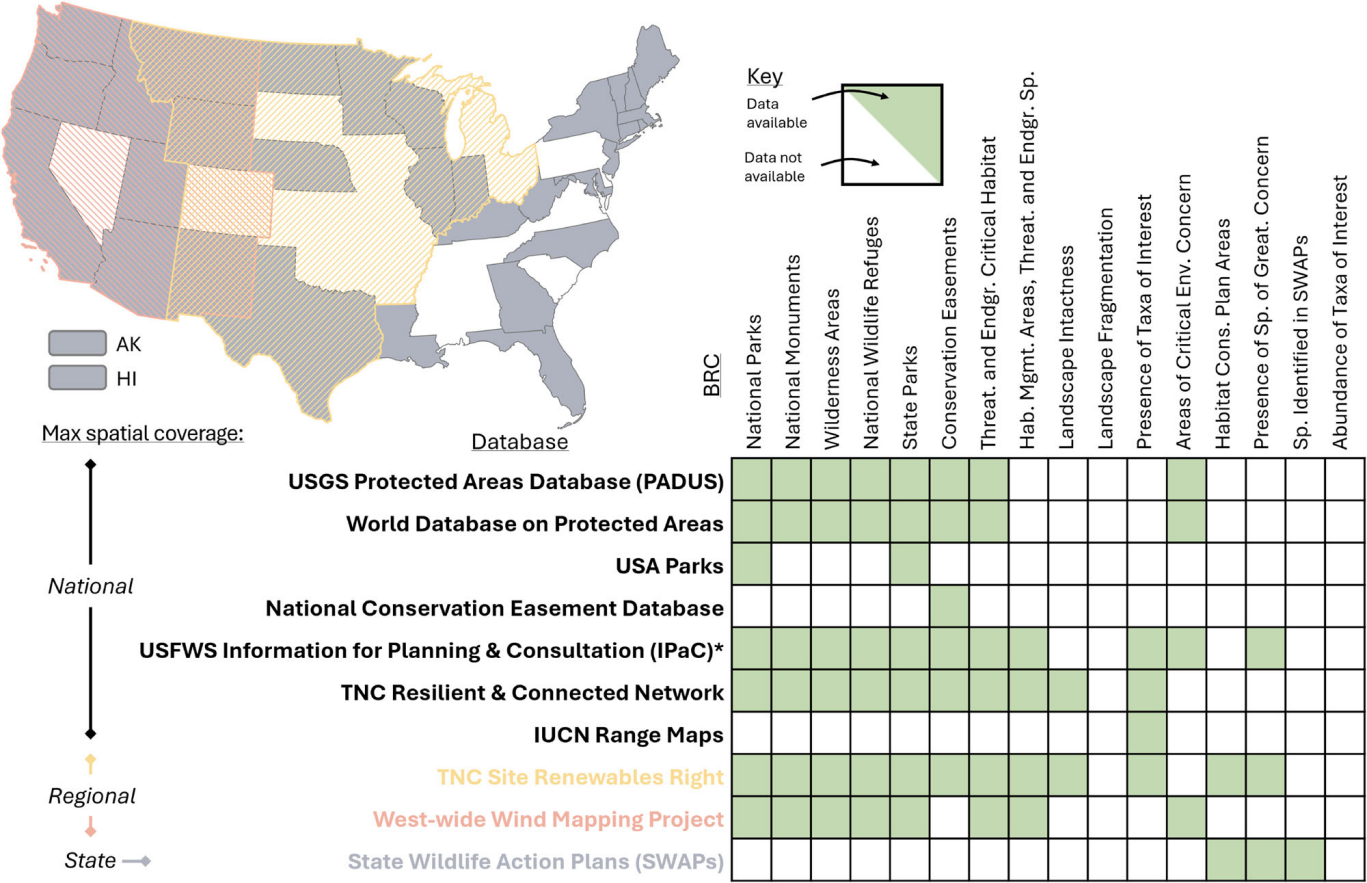
Core biodiversity-relevant criteria (BRCs) represented by practitioner-identified databases and their spatial extent. Green boxes indicate which BRCs are represented within the respective databases. Databases with data covering the US in its entirety are shown in black. The extent of regional databases—The Nature Conservancy Site Renewables Right project and the West-wide Wind Mapping Project—are mapped in yellow and pink, respectively. While all states have a wildlife action plan, those with open-access geographic information systems data are shown in gray. BRCs represented within State Wildlife Action Plans vary by state. Databases with an asterisk include geospatial data that are only downloadable to a pre-specified extent.

Practitioners highlighted ten repositories containing state-to-national level data across most core criteria (Fig. 3). Practitioners primarily identified the US Geological Survey Protected Areas Database (PADUS)^29^, World Database on Protected Areas^30^, USA Parks feature layer^31^, National Conservation Easement Database^32^, US Fish and Wildlife Service Information for Planning and Consultation^33^, The Nature Conservancy (TNC) Resilient and Connected Network^34^, and the International Union for the Conservation of Nature Red List Species Range Maps^35^ to contain core criteria data. Practitioners also offered useful regional resources, such as the TNC Site Renewables Right database^36^ or the West Wide Wind Mapping Project^37^, despite being more closely tailored to renewable energy development generally than LSS. Finally, practitioners suggested that State Wildlife Action Plans may also provide core criteria data, although such data vary by state.

The Information for Planning and Consultation database and TNC’s Resilient and Connected Network contain the most core BRCs of the databases (*n* = 11), followed by TNC’s Site Renewables Right Project (*n* = 9). The Information for Planning and Consultation site offers data integration from other sources, including the PADUS and National Conservation Easement Database. However, this dataset does not allow users to download the associated geospatial data for further analysis unless specifying a project site or polygon area (e.g., 750,000 mi^2^). Other identified datasets are also highly interconnected. For example, PADUS pulls data on protected areas and conservation easements from the World Database on Protected Areas and the National Conservation Easement Database, respectively. Data from PADUS are also used within the Information for Planning and Consultation database and TNC Resilient and Connected Network, indicating minimal data conflicts. Five of the six main databases identified were last updated as of 2019, except the National Conservation Easement Database providing data from 2017. As such, some of these databases may contain outdated geospatial data; we clarify the impact of this limitation below.

We identified 14 consensus and 2 near-consensus core biodiversity-relevant criteria used explicitly across the 13 US-based SSA-related studies (Supplementary Item 2). Summaries of all evaluated solar suitability analyses are provided in Supplementary Item 3. On average, the studies included less than half of the core criteria (*n* = 7.3). The studies that integrated the highest number of core BRCs were Wu et al.^38^ (*n* = 16), followed by Wu et al.^39^ (*n* = 15), while three studies integrated none of the core BRCs (e.g., refs. ^21,40,41^). The following core criteria were not covered by any study: land use history, landscape fragmentation, species identified in state wildlife action plans, and abundance and distribution of taxa of interest. Most studies integrated data on national parks, monuments, and wilderness areas (*n* = 10), primarily represented by the PADUS (*n* = 7). Nearly all other criteria were integrated within one-fourth of the studies examined. Importantly, many of these studies included other environmental criteria that did not fit our BRC classifications, which may have additional benefits for biodiversity, such as special designation areas denoted in Patankar et al.^42^.

### Core BRC and US regulations

Our analysis shows that US federal regulations primarily interact with core BRC from the first Delphi discussion round (Table 1). For some biodiversity-relevant criteria, such as conservation easements, the associated regulation may only apply under certain conditions. We highlight only federal legislation with an explicit written connection to each BRC, in alignment with their definitions provided in Supplementary Item 3.

**Table 1:** Core BRCs from Delphi rounds 1 and 2 and Associated US Federal Legislation

BRC	Federal Law
Round 1	
National parks	National Environmental Policy Act; Organic Act of 1916
State parks	National Environmental Policy Act; Organic Act of 1916
National monuments	National Environmental Policy Act; Antiquities Act of 1906
Wilderness areas	National Environmental Policy Act; Wilderness Act of 1964
National wildlife refuges	National Environmental Policy Act; National Wildlife Refuge System Administration Act of 1966
Conservation easements	National Environmental Policy Act^a^
Threatened and endangered critical habitat	National Environmental Policy Act; Endangered Species Act of 1973
Areas of critical environmental concern	Federal Land Policy and Management Act of 1976
Habitat conservation plan areas	National Environmental Policy Act^b^; Recovering America’s Wildlife Act (proposed, as of February 2025)
Round 2	
Presence of species of greatest conservation need	–
Presence of taxa of interest	–
Abundance of taxa of interest	–
Species identified in state wildlife action plans	–
Landscape intactness	–
Landscape fragmentation	–

Legislation provided was proposed as of the data collection period, August 2023. All except the Recovering America’s Wildlife Act were in effect as of 2025.

^a^The National Environmental Policy Act applies when federal funding or land management is involved.

^b^The National Environmental Policy Act applies if the conservation plan involves a federal agency.

## Discussion

Our findings reveal a subset of biodiversity-relevant criteria that are prioritized by practitioners for use within solar suitability analyses. First, practitioners consistently identified legally grounded criteria as core inputs for SSAs, clarifying their role as a screening baseline. Second, limited and inconsistent inclusion of non-statutory criteria, including landscape fragmentation, taxa of interest, and species identified in State Wildlife Action Plans, highlights how data availability and regulatory frameworks can shape BRC implementation within solar suitability analyses. Finally, criteria designated as peripheral were viewed as context-dependent or constrained by data resolution and scale, indicating that SSAs may adopt a tiered approach in which core criteria inform initial screening and peripheral criteria are applied during project-level evaluation. Our analysis highlights the potential for clearer distinction between BRC use driven primarily by regulatory compliance and concerns of broader biodiversity risk, as well as improved availability and accessibility of biodiversity data to support non-statutory BRCs. Addressing these needs could help practitioners better interpret SSA results, with direct implications for where large-scale solar is ultimately sited and the extent to which siting decisions avoid high-value biodiversity areas.

### Regulatory context shapes core criteria use

The core BRCs identified in Delphi Round 1 largely concerned compliance with federal environmental legislation—consistent with expectations and review of solar suitability analysis literature by Levin et al.^20^. Amid growing concerns over land-use and land-cover change and threats to biodiversity targets posed by LSS expansion^43,44^, biodiversity-relevant criteria perceived as relevant to solar planning relate sensibly to laws protecting critical biodiversity habitat. When conservation areas are not already excluded from large-scale solar buildout prospects, developers may avoid these spaces regardless to sidestep costly litigation, permit denial, or high compensatory mitigation costs^45^. This dynamic showcases how regulatory frameworks guide the inclusion of BRCs in solar suitability analyses and shape practical decision-making in large-scale solar development.

Considering the multidecadal lifetimes of LSS projects and their potential risks to local biodiversity^44,46^, solar suitability analyses using only legally enforceable biodiversity-relevant criteria may underrepresent current and forthcoming conservation spaces. Criticisms of US biodiversity protections highlight a mismatch between ambitious federal conservation goals (e.g., the 30-by-30 initiative) and the inadequacy of the existing protected areas network^47–50^. Out of 2218 imperiled species in the continental US, just under 10% occur mostly, if not entirely, within PADUS (public) conservation areas^49^. Conversely, over 13% of species’ modeled suitable habitat occurs completely outside protected areas, primarily across private lands in southwestern and southeastern states^47,49^. Pressure from human-driven land-use and land-cover change and climate-induced range shifts may exacerbate the capability of existing protected areas to harbor future species^48,51–53^. As such, solar suitability analyses including additional BRCs beyond those under current legal protections–including those relevant across private lands in the US–can provide a more comprehensive approach to avoiding sensitive areas.

Core criteria discerned from the second and third Delphi discussion rounds align less with federal law than those from the first round (Table 2). Instead, these criteria emphasize subnational legislation and conservation planning frameworks that may vary in strength among jurisdictions. To the extent that legal mechanisms set a baseline for biodiversity-relevant criteria use in SSAs^20^, states with a lower compliance threshold may include fewer criteria in siting assessments than those with more rigorous regulations. Protections for species and taxa of greatest conservation need illustrate this dynamic well. While the federal Endangered Species Act of 1973 regulates critical habitat and listed species, each state maintains its own regulatory framework for protecting at-risk species, often informed by State Wildlife Action Plans. These plans, developed under the State and Tribal Wildlife Grants Program, are required to identify and conserve species of greatest conservation need^54^. However, the criteria used to designate species of greatest conservation need are determined at the state level. The scope and legal strength of these protections vary substantially among states and are often more limited than those afforded under federal law. For example, in 2017, 32 states had legalized protections less extensive than the Endangered Species Act, of which 17 included the conservation of animals exclusively^55^. Hamilton et al.^56^ posits that variation in State Wildlife Action Plan strategies amongst neighboring states instigates conservation challenges; instead, regional or multistate networks protecting species of greatest conservation need may be necessary. As such, the legal protections for species outside of Endangered Species Act jurisdiction extend only to the regulations supporting State Wildlife Action Plans. So far as legal compliance encourages BRC use in solar suitability analyses, the rigor of biodiversity risk assessments in SSAs may be uneven across jurisdictions.

**Table 2:** Biodiversity-related categories and criteria identified by Levin et al.^20^

Protected and unprotected important areas for biodiversity	Land cover	Vegetation	Landscape intactness	Ecosystem services
Protected areas	Forests	Vegetation	Landscape intactness	Ecosystem services
Important bird areas/bird sanctuaries/flyways	Wetlands	Normalized difference vegetation index		
Important biodiversity areas				
World heritage sites				
Biosphere reserves				

Bolded headers in the above table refer to categories of biodiversity-related criteria, while the subgroups comprise the criteria themselves.

### Inconsistent and unavailable data inhibit the use of core criteria

The PADUS was favored by SSA-related studies to depict core biodiversity-relevant criteria, particularly those related to protected areas. While this database offers an open-source, robust representation of BRCs associated with legal compliance, it fails to account for half of the non-statutory full-consensus core BRCs. Solely using core biodiversity-relevant criteria from the PADUS may disproportionately assess essential biodiversity spaces by relying on criteria that are inexpensive and straightforward to integrate. Additional data related to sub-national biodiversity conservation, such as that found within State Wildlife Action Plans or state-led geospatial hubs, could inform core BRC distribution not covered by the PADUS. Only four SSA-related studies–that of Katkar et al.^57^, Wu et al.^58^, Wu et al.^38^, and Majumdar and Pasqualetti^59^—supplemented the PADUS with data from such databases, however. Assessments relying on biodiversity-relevant criteria from the PADUS without aid from additional datasets may also critically impair thorough biodiversity evaluations due to limited data on habitat management areas, landscape intactness, landscape fragmentation, presence and abundance of taxa of interest, presence of species of greatest conservation need, and species identified within State Wildlife Action Plans.

Protections and planning for species of greatest conservation need can pose additional financial and temporal challenges for solar suitability analyses, particularly those conducted by research institutions that may not have access to sensitive ecological data. Sensitive data, such as species distribution, abundance, or habitat, may be publicly restricted or of coarse spatial resolution to limit human interference. NatureServe (https://explorer.natureserve.org/) offers these data for free and paid subscribers, and at varying levels of spatial precision. Of the 80 taxonomic groups and species identified by the US Department of Energy as affected by solar development^60^, all but one have publicly accessible distribution data represented in NatureServe, typically mapped using 343-mi^2^ hexagonal grids. NatureServe also mediates access to precise state-level natural heritage data, but often with additional financial stipulations. For example, in Mississippi, geospatial data requests on rare species distributions start at $45 per quadrangle and require landowner permission^61^. Comprising 133 quadrangles mostly in private land ownership, acquiring such data on a state scale would present a costly and timely challenge for SSA administrators, despite the potential for these data to inform key areas of avoidance for LSS development. As a result, SSAs may proceed with coarse-resolution data or without including species distribution-related BRCs to bypass these barriers, leading to assessments that may underrepresent biodiversity impacts. In other instances, large-scale solar development may be directed toward states with deficiencies in data or regulations (e.g., those with weaker ecologically restrictive siting constraints), which may still harbor considerable biodiversity. To this end, partnerships with state agencies may be vital in facilitating access to data that improve the comprehensiveness and accuracy of biodiversity-relevant criteria.

Landscape fragmentation and intactness, two core biodiversity-relevant criteria, present critical yet underutilized metrics for aligning conservation efforts and energy development. Landscapes connecting protected areas are essential in sustaining biodiversity^62^, yet conversion by LSS can impede animal movement^63^. This struggle is principally waged across US private lands, which host the majority of priority connectivity channels and existing solar sites^47,62,64^. At the state level, where many landscape connectivity initiatives arise, conservation efforts have emphasized funding some wildlife interconnection projects rather than protecting swathes of intact habitat^65^. It is then generally unclear, from a data standpoint, where the most salient stretches of intact habitat exist and thereby could be integrated as exclusion zones within SSAs. Consequently, many spaces may remain vulnerable to fragmentation by large-scale solar.

Several studies have modeled priority zones for maintaining landscape connections^62,66^, or employ metrics like the human modification index to calculate intactness^67^; however, there remains a notable lack of national-scale public data explicitly focused on landscape fragmentation. This gap is reflected in many existing US-based SSA-related studies, echoing the difficulty of incorporating landscape integrity into solar siting decisions. A handful of resources offers land cover change data^68,69^, but requires additional calculations to isolate fragmented landscapes, such as identifying the inverse of intact or connected landscapes. Thorough assessments of fragmented landscapes may be limited by current data availability, a lack of standardized methodologies, and the absence of broad legislative frameworks supporting their integration. Such evaluations are increasingly imperative to plan for substantial projected overlap between species’ climate-driven range shifts and LSS development^70^.

### Tradeoffs in data inclusion shape SSA outcomes

Open-access databases suggested by practitioners highlight a challenge for applying BRCs within solar suitability analyses: tradeoffs among cost, timeliness, spatial resolution, and analytical feasibility. While open-access databases may provide a quick and low-cost data option, inconsistent or infrequent data updates reduce the capacity for these databases to adequately represent constant changes in ecology, land use, and conservation boundaries. For example, the rapidly growing scope of protected areas within the 30-by-30 initiative (a former US conservation goal to conserve at least 30% of lands and waters by 2030, formalized through Executive Order 14008 and supported by state conservation programs, despite being officially rolled back in 2025^71^) may not be well reflected in the PADUS, which is updated biennially. Similarly, other databases developed from single foundational studies (e.g., the West Wide Wind Mapping Project, TNC studies) risk becoming outdated over time, leading to SSAs that may misrepresent species distributions or conservation boundaries. Regular updates require significant financial and temporal investment by host organizations; however, these investments could improve the accuracy of solar suitability analyses. When the accuracy of these datasets cannot be improved, greater transparency regarding data timeliness and relevance could support more informed use. Sharing dynamic and accurate data is especially critical for LSS planning, which must anticipate changing biodiversity patterns and protections over multidecadal projects’ lifetimes.

Practitioners’ designations of peripheral criteria showcase how data scale shapes also BRC use in solar suitability analyses. Peripheral criteria identified in this study tend to reflect broader ecological attributes, including habitat context, microclimate diversity, and proximity-based relationships, which are often context-dependent and less directly tied to standardized, landscape-scale datasets. During the Delphi discussion, participants noted that such criteria—while necessary for fully evaluating solar suitability—require site-scale data collection, leading to their classification as peripheral. Limited funding and time for proactive data collection hinder the feasibility of using peripheral criteria in all SSAs. Instead, core BRCs can support broad, screening-level suitability assessments, with peripheral criteria applied to help narrow or refine suitable zones during project-level evaluations. Integrating peripheral criteria as a fine-scale filter may more accurately represent exclusion areas or alter the relative weight of ecological risks than exclusively using core biodiversity-relevant criteria in SSAs; however, peripheral data must be updated and available at the SSA scale. Future research may explore whether access to fine-resolution data at scale would reclassify peripheral BRCs to a core designation.

Interestingly, practitioners designated some biodiversity-relevant criteria with available geospatial data, such as surface hydrology, proximity to forests, or soil properties, as peripheral. While this may reflect the limited, site-level significance of these BRCs, it is also plausible that practitioners perceived them as less critical to biodiversity considerations in solar suitability analyses. In disseminating the Delphi focus group, one participant questioned the inclusion of some BRCs altogether, suggesting that they may not warrant even a peripheral designation. This criticism alludes to more profound questions about acceptable levels of environmental impact and risk from LSS development. At what point do SSAs overly-scrutinize BRC risks? And, by extension, when does large-scale solar development balance acceptable biodiversity trade-offs between global benefits from avoided carbon and the negative consequences on local ecosystems? These questions provide fodder for further inquiry as land for renewable energy and biodiversity alike becomes increasingly scarce.

### Improving SSAs with community input and data availability

Our evaluation provides a baseline for biodiversity-relevant criteria inclusion in SSAs, which can be enhanced through contextualization and input from stakeholders. Collaborating with local entities within the SSA study areas can help assess community preferences for trade-offs between local biodiversity impacts and the broader benefits generated by LSS. In this sense, stakeholders may participate more directly in defining solar suitability as tied to the socio-ecological context of the impacted region. Moreover, the local knowledge from these groups may inform the appropriateness of peripheral BRCs' use, given the context of the area and data availability. Partnerships with sub-national conservation entities, including universities and research institutes, may provide access to high-resolution geospatial data on BRCs. Future work could illustrate how energy and biodiversity priorities vary across geographies, given the diversity in BRCs used in solar suitability analyses.

Increasing the availability of and access to biodiversity data remains another frontier for improving SSAs. Several core and peripheral criteria identified in this study, including species distributions and landscape connectivity, are not consistently represented in widely used open-access datasets, limiting their integration into screening-level analyses. Partnerships with agencies and organizations involved in environmental review and conservation planning may help address data gaps and access constraints, particularly where relevant datasets are not publicly available. Additionally, standardizing biodiversity monitoring and assessment metrics across existing LSS sites may also support the development of more robust, site-scale datasets^72,73^ and improve the consistency of Biodiversity-Relevant Criterion application across projects. More research is needed to identify priority data gaps and develop standardized methods and metrics for generating biodiversity data to support consistent BRC application in SSAs.

### Global and national outlooks

**Fig. 4:**
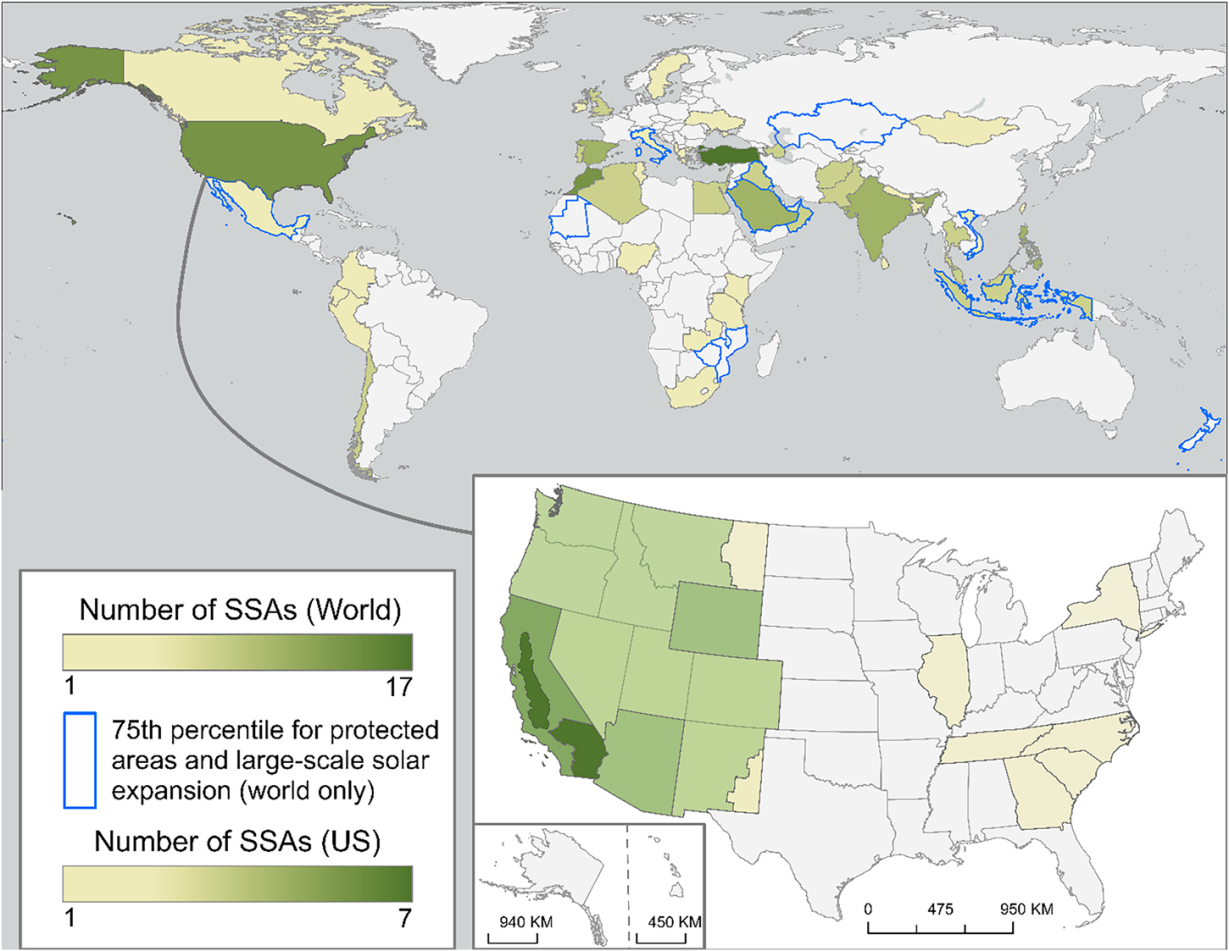
Solar suitability analysis-related studies identified by Levin et al.^20^ on global and United States scales. Yellow-to-green gradients indicate low-to-high solar suitability analysis counts per geographic region, respectively. Counts reflect data collected in 2023. Countries falling into the 75th percentiles of both large-scale solar buildout (Global Energy Monitor^78^, capacity in megawatts) and protected areas (UNEP-WCMC and IUCN^79^, total area) are shown with a blue border. Protected areas represent nationally and internationally designated areas reported to the World Database on Protected Areas, including a range of designation types (e.g., national parks, nature reserves, and other conserved areas), aggregated across countries. While protected area classifications and governance types vary among countries, this dataset provides a globally consistent proxy for conservation priorities.

Although this study focuses on US-based expertise, our results have broad relevance to SSAs of any geographical context. Our findings highlight a generalizable pattern in the prioritization of biodiversity-relevant criteria shaped by regulatory frameworks and the availability of spatially explicit data, which may help inform the identification of useful BRCs within other national contexts. Many countries are grappling with the expansion of LSS while also recognizing the need to safeguard native biodiversity. Some regions—such as Mexico, Italy, New Zealand, Mauritania, Kazakhstan, Mozambique, and Zimbabwe—have significant areas dedicated to both land uses but lack established SSAs to guide development (Fig. 4). Countries with high biodiversity and projected LSS buildout, such as Brazil and China, are expected to face overlap between priority landscapes for conservation and solar, necessitating careful consideration of how to balance both land uses^74^. Differences among international conservation legislation, such as the existence of protected areas, varying levels of acceptable developmental intensities, and national conservation priorities, may partially influence the suitability of LSS development locations. Proactive solar suitability analyses in biodiversity and LSS hotspot regions may benefit from international databases, such as the World Database on Protected Areas, to prioritize conservation areas within their legal frameworks. However, the limited coverage of core BRCs in this database (Fig. 3) indicates that additional data, such as that for landscape fragmentation and the presence of taxa of interest, may be needed to support comprehensive SSAs. Further work is needed to identify which biodiversity-relevant criteria are most appropriate within other national contexts.

Spatial gaps exist in the coverage of SSA-related studies within the US. As of 2023, much of the eastern half of the country lacked SSA-related studies, while certain regions in the western US had as many as seven overlapping studies (Fig. 4). Despite some deficiencies in SSA-related studies, nearly every state had adopted one LSS project by 2023^75^. This mismatch suggests that solar development in understudied regions may proceed without adequate consideration of conservation priorities or the best available ecological data. Further exploration into Delphi-style BRC identification can help address this gap, specifically tailored to SSA-deficient regions and with practitioners who hold region-specific roles in LSS development and biodiversity conservation. This approach would enhance the representation of biodiversity conservation priorities and improve the integration of the best available ecological data into LSS siting decisions.

### Study limitations

Two key study limitations warrant further discussion. First, we share practitioner perceptions from a relatively small sample (*n* = 15). While this size was effective for a facilitated focus group setting, it lacks the diversity of a larger cohort. Similarly, this small sample size prohibits assumptions of regional representation, despite efforts to ensure practitioners’ regional diversity. Some criteria designations, particularly those in near-consensus, may be misrepresented due to this study's limitation. Future research should reevaluate our findings with a larger sample size or a region-specific assessment.

Second, modifying the Delphi method to better foster group discussion may have inadvertently inhibited individual expression. Practitioners may have felt the need to conform to group majority opinions to meet discussion time limits or avoid conflict with others. Consequently, this could create feigned group consensus, skewing some biodiversity-relevant criteria designations. Additional review rounds incorporating anonymous feedback could reveal increased disagreement amongst BRC designations and address this challenge in a future study.

These limitations, while worth discussing, do not detract from the main findings of this work. Instead, they highlight opportunities for future research to reevaluate and contribute to our initial assessment of BRCs in solar suitability analyses. Addressing these challenges could further refine the essential criteria for SSAs as finding space for solar deployment and biodiversity conservation becomes increasingly paramount.

## Methods

**Fig. 5:**
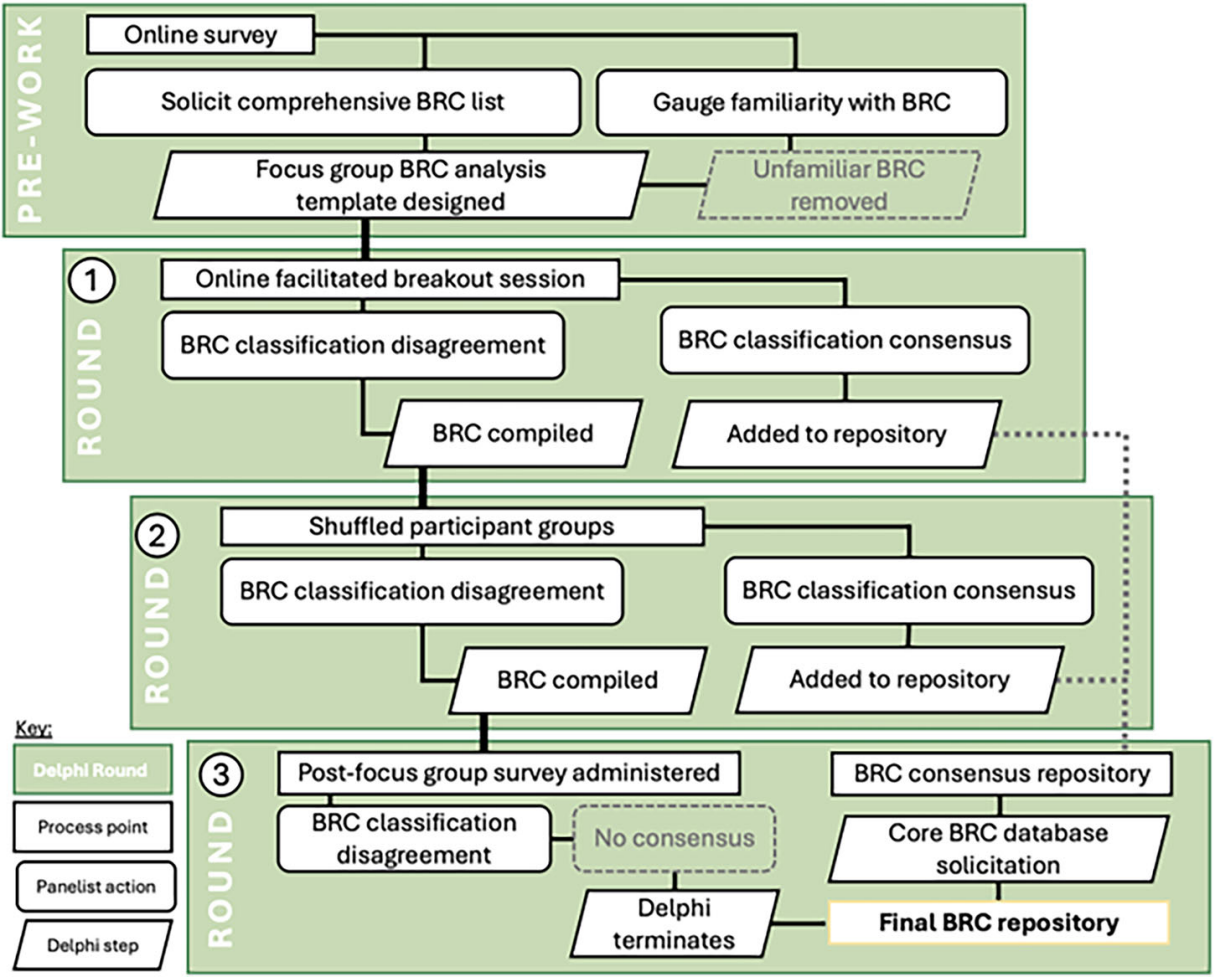
Delphi technique flow diagram. The Delphi technique is a structured solicitation method involving multiple rounds of participant review to ultimately produce consensus on a subject matter. This study modifies the traditional Delphi method from fully anonymous to semi-anonymous, generating intra-group discussion on complex biodiversity topics. The objective of this process was to identify biodiversity-relevant criteria that should be used in every solar suitability assessment as agreed upon by the entirety of our practitioner panel. Dotted lines indicate flows separate from the main procedure process (solid lines).

We used a mixed-methods approach to identify and evaluate Biodiversity Relevant Criteria. In doing so, we held a modified Delphi-style focus group (Fig. 5), established a repository of core and peripheral criteria, and conducted a literature review of US-based SSA-related studies. The Delphi process collected both quantitative classifications (e.g., sorting biodiversity-relevant criteria into core or peripheral categories) and qualitative input (e.g., suggestions for new criteria and datasets). We adopted the Delphi technique because it is widely used to solicit structured expert input on complex or data-limited topics, such as biodiversity planning for large-scale solar development. While the primary analysis presented here is quantitative, qualitative responses were reviewed to capture emerging themes and clarify participant rationales. The goals of these processes were to establish a baseline for BRCs' use in future solar suitability analyses and inform gaps where additional research would aid conservation planning within SSAs. The Delphi approach was especially well-suited for this work because BRC selection involves subjective decision-making and, in many cases, practitioner knowledge that cannot be easily obtained through published data sources alone.

### Online survey

We first organized a panel with practitioners from the US holding expertise or experience with solar and/or biodiversity from academic, governmental, and NGO backgrounds (*n* = 15). Of this group, most had contributed to applied processes relevant to solar siting, including environmental assessment, biodiversity evaluation, or the development of solar suitability analyses. We intentionally selected practitioners from the US so that relationships among BRC, their priority, and policy could be constrained to a single regulating body. We identified and contacted some initial panel members as practitioners from Condon et al.^76^, interested in participation in similar research. Practitioners’ knowledge covered all major contiguous US regions, including the Pacific Northwest, Southwest, Midwest, Southeast, and Northeast regions of the US. As part of our selection process, we ensured that each region was represented by at least one practitioner. Due to the limited pool of individuals working on the subject matter identified by Condon et al.^76^, we supplemented this group with known practitioners who compensated for deficiencies in the group’s overall expertise background or regional knowledge.

Practitioners first completed an online survey to (a) gauge familiarity with SSAs and (b) identify which biodiversity-relevant criteria should be included in the Delphi review process. We evaluated familiarity with SSAs on a 5-point Likert scale (“not at all familiar” to “very familiar”), and practitioners self-identified their expertise in groups of BRCs from Levin et al.^20^ (Table 2). For (b), practitioners indicated which BRCs they believed were and were not used in SSAs, as well as potential barriers to including BRCs in solar suitability analyses. Any new BRCs identified in the survey but not present in the literature review were compiled for the focus group. Surveys were anonymous to encourage candid responses. Survey responses indicated that participants generally reported moderate to high familiarity with SSAs and identified a consistent set of commonly used biodiversity-relevant criteria, which informed the selection and refinement of criteria included in the subsequent Delphi process. The findings from this survey are presented in Levin et al.^20^.

### Delphi-style focus group

On August 4th, 2023, we held a virtual conference focus group to best accommodate the geographical diversity of practitioners. We dedicated the first 15 min to a short presentation on the biodiversity-relevant criteria identified within the literature review (Levin et al.^20^) to clarify baseline assumptions of existing BRCs in solar suitability analyses. This presentation included working definitions for all BRCs examined in this analysis (Supplementary Item 1).

Following the presentation, we conducted the first round of Delphi analysis by dispersing practitioners into 5 facilitated breakout rooms, each with 3 practitioners. We aggregated the practitioners into these groups to balance individual thought and group conformity pressure, the latter of which can skew results unfavorably^77^. Each breakout group was given access to a virtual Jamboard slide. Jamboard (https://support.google.com/jamboard/answer/7424836?hl=en) was an online platform allowing real-time group member collaboration, which has since been decommissioned as of January 2025. Using Jamboard, practitioners collaboratively sorted pre-survey and literature-derived BRCs into “core” (essential to all SSAs) or “peripheral” (data- or site-scale limited) categories. Participants were instructed to distinguish between these categories based on applicability across SSAs and feasibility constraints (i.e., data availability and scale), although classification decisions may also have reflected perceived relevance to SSA decision-making. Groups could add new biodiversity-relevant criteria not previously identified, but could not remove existing ones, as the focus was on prioritization. The sorting process lasted approximately 1 h. While this section was not anonymous to the participants within the breakout rooms, the breakout room consensus was anonymous to other groups.

After completing the first Delphi deliberation round, practitioners took a 15-min break while the authors compiled and re-sorted the data for round two. Practitioners viewed the criteria in which all breakout rooms achieved sorting consensus as core or peripheral before the next Delphi round. For the second sorting round, practitioners were intentionally paired up with those they had not previously spoken with to generate novel ideas and encounter differing opinions that may influence sorting. The breakout rooms received a new Jamboard slide of disputed (<100% overall consensus in round 1) BRCs, and practitioners re-sorted criteria into core and peripheral categories. For this round, groups were not permitted to offer new biodiversity-relevant criteria. Practitioners took another hour to discuss and re-categorize the BRCs. This was the final round of the focus group evaluation.

### Focus group post-work

After the focus group, we distributed an anonymous survey to (a) conduct a final re-categorization of disputed BRCs, (b) collect datasets for agreed-upon core criteria, and (c) gather feedback on the process. Dataset contributions included links to existing sources, descriptions of how missing data could be collected, or notes on unreliability. The purpose of collecting data links was to evaluate whether limited data inhibited the utility of core criteria.

### Data dissemination

Results from all Delphi rounds were aggregated by Biodiversity-Relevant Criterion and level of consensus. Criteria with 100% agreement were classified as “in consensus,” while those with 80–90% were classified as “near consensus.” Criteria with less than 80% agreement were classified as “in disagreement.” While full consensus is optimal, it is challenging to achieve in multi-stakeholder assessments; therefore, our analysis progressed with consensus and near-consensus criteria.

We compared our final BRC cohort to the BRCs and databases used in the US-based SSA-related studies identified by Levin et al.^20^, and an additional study that was overlooked by this review. Importantly, we limited our comparison to studies assessing general landscape suitability for LSS rather than those focused on a preselected project location, which may involve proprietary data or modeling. Our evaluation of US-based SSA-related studies may serve as a precursor to further project-specific assessments; however, such specific assessments were outside the scope of this examination.

### Comparison of core BRCs to US federal regulations

Finally, we analyzed connections between identified core biodiversity-relevant criteria and relevant US federal regulations, sparked by a theorized relationship from Levin et al.^20^. Levin et al.^20^ proposed that BRCs embedded within federal regulatory frameworks are more likely to be consistently included in solar suitability analyses, indicating that regulatory compliance can drive criteria selection. The lead author of the current study reviewed federal regulations proposed as of 2023 that could protect, govern, or otherwise influence the core BRCs identified by the focus group. We included regulations current through that year to reflect the standards in place at the time of the focus group. Practitioners were invited to review the list of regulations to ensure accurate representation of core BRCs.

## Supplementary information

Supplementary information

## Data Availability

The datasets generated and/or analyzed during the current study are available in the figshare repository (10.6084/m9.figshare.28447754). Only datasets with downloadable geospatial formats (e.g., raster, vector, geodatabase, CSV) and a documented last update date were included. This ensures future SSA practitioners can directly access current, accessible, and spatially explicit biodiversity data. Other data on BRC definitions and SSA-related studies can be found in the Supplementary Materials 1–3.

## References

[1] International Energy Agency. Renewables 2024, Paris, 2024. Available: https://www.iea.org/reports/renewables-2024.

[2] U.S. Energy Information Administration. U.S. Short-Term Energy Outlook. Available: https://www.eia.gov/outlooks/steo/pdf/steo_full.pdf (2025).

[3] The Brattle Group. Comparing the costs of utility-scale and residential-scale PV. Available: https://www.brattle.com/wp-content/uploads/2017/10/7626_comparing_the_costs_of_utility-scale_and_residential-scale_pv_-_factsheet.pdf (2017).

[4] U.S. Energy Information Administration. Utility-scale U.S. solar electricity generation continues to grow in 2024. Available: https://www.eia.gov/todayinenergy/detail.php?id=63324.

[5] Ardani, K. et al. *Solar Futures Study* (Solar Energy Technologies Office, U.S. Department of Energy, 2021).

[6] Haines-Young, R. Land use and biodiversity relationships. *Land Use Policy* **26**, S178–S186 (2009).

[7] Ellis, E. C., Klein Goldewijk, K., Siebert, S., Lightman, D. & Ramankutty, N. Anthropogenic transformation of the biomes, 1700 to 2000. *Glob. Ecol. Biogeogr.* **19**, 589–606 (2010).

[8] Newbold, T. et al. Global effects of land use on local terrestrial biodiversity. *Nature* **520**, 45–50 (2015). 25832402 10.1038/nature14324

[9] Tilman, D. et al. Future threats to biodiversity and pathways to their prevention. *Nature* **546**, 73–81 (2017). 28569796 10.1038/nature22900

[10] Ellis, E. C. et al. People have shaped most of terrestrial nature for at least 12,000 years. *Proc. Natl. Acad. Sci. USA* **118**, e2023483118 (2021). 33875599 10.1073/pnas.2023483118 PMC8092386

[11] Al Garni, H. Z. & Awasthi, A. Solar PV power plants site selection: a review. in *Advances in Renewable Energies and Power Technologies* (ed. Yahyaoui, I.) Ch 2, 57–75 (Elsevier, 2018).

[12] Scherer, L. et al. Biodiversity impact assessment considering land use intensities and fragmentation. *Environ. Sci. Technol.* **57**, 19612–19623 (2023). 37972360 10.1021/acs.est.3c04191 PMC10702493

[13] Simkin, R. D., Seto, K. C., McDonald, R. I. & Jetz, W. Biodiversity impacts and conservation implications of urban land expansion projected to 2050. *Proc. Natl. Acad. Sci. USA* **119**, e2117297119 (2022). 35286193 10.1073/pnas.2117297119 PMC8944667

[14] Kehoe, L. et al. Global patterns of agricultural land-use intensity and vertebrate diversity. *Divers. Distrib.* **21**, 1308–1318 (2015).

[15] Carlisle, J. E., Kane, S. L., Solan, D., Bowman, M. & Joe, J. C. Public attitudes regarding large-scale solar energy development in the U.S. *Renew. Sustain. Energy Rev.* **48**, 835–847 (2015).

[16] Ioannidis, R. & Koutsoyiannis, D. A review of land use, visibility and public perception of renewable energy in the context of landscape impact. *Appl. Energy* **276**, 115367 (2020).

[17] Mulvaney, D. Identifying the roots of Green Civil War over utility-scale solar energy projects on public lands across the American Southwest. *J. Land Use Sci.* **12**, 493–515 (2017).

[18] Susskind, L. et al. Sources of opposition to renewable energy projects in the United States. *Energy Policy* **165**, 112922 (2022).

[19] National Caucus of Environmental Legislators. 30×30 midpoint: are States on track to conserve 30% of land and waters by 2030? Available: https://www.ncelenviro.org/articles/30x30-midpoint-are-states-on-track-to-conserve-30-of-land-and-waters-by-2030/.

[20] Levin, M. O. et al. Bibliographic synthesis of biodiversity-relevant criteria for solar energy siting. *Renew. Sustain. Energy Rev.* **223**, 116026 (2025).

[21] Kwak, Y., Deal, B. & Heavisides, T. A large scale multi criteria suitability analysis for identifying solar development potential: a decision support approach for the state of Illinois, USA. *Renew. Energy* **177**, 554–567 (2021).

[22] Charabi, Y. & Gastli, A. PV site suitability analysis using GIS-based spatial fuzzy multi-criteria evaluation. *Renew. Energy* **36**, 2554–2561 (2011).

[23] Stoms, D. M., Dashiell, S. L. & Davis, F. W. Siting solar energy development to minimize biological impacts. *Renew. Energy* **57**, 289–298 (2013).

[24] Bureau of Land Management. Approved resource management plan amendments/ record of decision (ROD) for solar energy development in six southwestern states. Available: https://blmsolar.anl.gov/documents/docs/peis/Solar_PEIS_ROD.pdf (2021).

[25] Gacu, J. G., Garcia, J. D., Fetalvero, E. G., Catajay-Mani, M. P. & Monjardin, C. E. F. Suitability analysis using GIS-based analytic hierarchy process (AHP) for solar power exploration. *Energies* **16**, 6724 (2023).

[26] United States Department of State and United States Executive Office of the President. The long-term strategy of the United States: pathways to net-zero greenhouse gas emissions by 2050. Available: https://www.whitehouse.gov/wp-content/uploads/2021/10/us-long-term-strategy.pdf.

[27] NatureServe. Biodiversity in focus: United States edition. Arlington, VA, 2023. Available: https://www.natureserve.org/bif.

[28] Mukherjee, N. et al. The Delphi technique in ecology and biological conservation: applications and guidelines. *Methods Ecol. Evol.* **6**, 1097–1109 (2015).

[29] U.S. Geological Survey. Protected areas database of the United States (PAD-US) 2.1 - World Database on Protected Areas (WDPA) Submission (ver 1.1, April 2021). U.S. Geological Survey. 10.5066/P9IVLRSS. 2021.

[30] UNEP-WCMC. Protected Area Profile for United States of America from the World Database on Protected Areas. Available: https://www.protectedplanet.net/country/USA.

[31] ESRI. USA Parks. Available: https://www.arcgis.com/home/item.html?id=e49e181ac82c46edac3ae601ebb3ef2d (2024).

[32] Ducks Unlimited and The Trust for Public Land. National conservation easement database. Available: https://www.conservationeasement.us/downloads/ (2024).

[33] U.S. Fish and Wildlife Service, Information for Planning and Consultation. Available: https://ipac.ecosphere.fws.gov/.

[34] The Nature Conservancy. The Nature Conservancy resilient and connected network. Available: https://www.conservationgateway.org/ConservationPractices/ClimateChange/Pages/RCN-Downloads.aspx (2024).

[35] International Union for the Conservation of Nature. Red list of threatened species. Available: https://www.iucnredlist.org/resources/spatial-data-download (2024).

[36] The Nature Conservancy. Site renewables right: accelerating a clean and green renewable energy buildout in the Central United States. The Nature Conservancy’s Great Plains Renewable Energy Initiative. Available: http://www.nature.org/siterenewablesright (2024).

[37] Sullivan, R. & Lopez, J. *West-Wide Wind Mapping Project Report*. Available: https://wwmp.anl.gov/report/wwmp-project-report.pdf (2016).

[38] Wu, G. C. et al. Minimizing habitat conflicts in meeting net-zero energy targets in the western United States. *Proc. Natl. Acad. Sci. USA* **120**, e2204098120 (2023). 36656853 10.1073/pnas.2204098120 PMC9942791

[39] Wu, G. C. et al. Low-impact land use pathways to deep decarbonization of electricity. *Environ. Res. Lett.* **15**, 074044 (2020).

[40] Brewer, J., Ames, D. P., Solan, D., Lee, R. & Carlisle, J. Using GIS analytics and social preference data to evaluate utility-scale solar power site suitability. *Renew. Energy* **81**, 825–836 (2015).

[41] Rebecca, H., Santini, R. & Brownson, J. R. S. “GIS-Based Spatial Analysis For Large-Scale Solar Power And Transmission Line Issues: Case Study Of Wyoming, U.S.” Paper presented at 41st American Solar Energy Society Meeting. Proceedings of the 41st American Solar Energy Society Meeting (2012). https://www.researchgate.net/publication/262566355_GIS-based_Spatial_Analysis_For_Large-Scale_Solar_Power_And_Transmission_Line_Issues_Case_Study_Of_Wyoming_US.

[42] Patankar, N., Sarkela-Basset, X., Schivley, G., Leslie, E. & Jenkins, J. Land use trade-offs in decarbonization of electricity generation in the American West. *Energy Clim. Change* **4**, 100107 (2023).

[43] Hernandez, R. R., Hoffacker, M. K., Murphy-Mariscal, M. L., Wu, G. C. & Allen, M. F. Solar energy development impacts on land cover change and protected areas. *Proc. Natl. Acad. Sci. USA* **112**, 13579–13584 (2015). 26483467 10.1073/pnas.1517656112 PMC4640750

[44] Hernandez, R. R. et al. Environmental impacts of utility-scale solar energy. *Renew. Sustain. Energy Rev.* **29**, 766–779 (2014).

[45] Dashiell, S., Buckley, M. & Mulvaney, D. Green light study: economic and conservation benefits of low-impact solar siting in California. Available: https://www.nature.org/content/dam/tnc/nature/en/documents/FINAL_Green_Light_Report_LR.pdf (2019).

[46] Walston, L. J., Rollins, K. E., LaGory, K. E., Smith, K. P. & Meyers, S. A. A preliminary assessment of avian mortality at utility-scale solar energy facilities in the United States. *Renew. Energy* **92**, 405–414 (2016).

[47] Jenkins, C. N., Van Houtan, K. S., Pimm, S. L. & Sexton, J. O. US protected lands mismatch biodiversity priorities. *Proc. Natl. Acad. Sci. USA* **112**, 5081–5086 (2015). 25847995 10.1073/pnas.1418034112 PMC4413281

[48] Dietz, M. S., Belote, R. T., Gage, J. & Hahn, B. A. An assessment of vulnerable wildlife, their habitats, and protected areas in the contiguous United States. *Biol. Conserv.* **248**, 108646 (2020).

[49] Hamilton, H. et al. Increasing taxonomic diversity and spatial resolution clarifies opportunities for protecting US imperiled species. *Ecol. Appl.* **32**, e2534 (2022). 35044023 10.1002/eap.2534 PMC9286056

[50] Dreiss, L. M. & Malcom, J. W. Identifying key federal, state, and private lands strategies for achieving 30 × 30 in the United States. *Conserv. Lett.* **15**, e12849 (2022).

[51] Belote, R. T. et al. Mapping conservation strategies under a changing climate. *BioScience* **67**, 494–497 (2017). 28584341 10.1093/biosci/bix028 PMC5451290

[52] Hoffmann, S., Irl, S. D. H. & Beierkuhnlein, C. Predicted climate shifts within terrestrial protected areas worldwide. *Nat. Commun.* **10**, 4787 (2019). 31636257 10.1038/s41467-019-12603-w PMC6803628

[53] Dreiss, L. M. et al. Targeting current species ranges and carbon stocks fails to conserve biodiversity in a changing climate: opportunities to support climate adaptation under 30 × 30. *Environ. Res. Lett.* **17**, 024033 (2022).

[54] Association of Fish and Wildlife Agencies. State wildlife action plans. Available: https://www.fishwildlife.org/afwa-informs/state-wildlife-action-plans.

[55] Camacho, A. E., Robinson-Dorn, M. J., Yildiz, A., Teegarden, T. *Assessing State Laws and Resources for Endangered Species Protection*, 3060882 (Social Science Research Network, 2017).

[56] Hamilton, H., Rapacciuolo, G., Kanter, J., Jones-Farrand, D. T. & Young, B. E. A landscape conservation perspective of state species of greatest conservation Need. *Conserv. Sci. Pract.* **6**, e13223 (2024).

[57] Katkar, V. V., Sward, J. A., Worsley, A. & Zhang, K. M. Strategic land use analysis for solar energy development in New York State. *Renew. Energy* **173**, 861–875 (2021).

[58] Wu, G. C., Torn, M. S. & Williams, J. H. Incorporating land-use requirements and environmental constraints in low-carbon electricity planning for California. *Environ. Sci. Technol.* **49**, 2013–2021 (2015). 25541644 10.1021/es502979v

[59] Majumdar, D. & Pasqualetti, M. J. Dual use of agricultural land: introducing ‘agrivoltaics’ in Phoenix Metropolitan Statistical Area, USA. *Landsc. Urban Plan.* **170**, 150–168 (2018).

[60] U.S. Department of Energy. Solar impacts on wildlife and ecosystems: request for information response summary, DE-FOA-0002583. Available: https://www.energy.gov/sites/default/files/2021-11/Solar%20Impacts%20on%20Wildlife%20and%20Ecosystems%20Request%20for%20Information%20Summary.pdf (2021).

[61] Mississippi Wildlife, Fisheries, & Parks. Request natural heritage information. Available: https://www.mdwfp.com/ms-museum-nature-science/mississippi-natural-heritage-program/request-natural-heritage-information.

[62] Belote, R. T. et al. Identifying corridors among large protected areas in the United States. *PLoS ONE* **11**, e0154223 (2016). 27104683 10.1371/journal.pone.0154223 PMC4841590

[63] Levin, M. O. et al. Solar energy-driven land-cover change could alter landscapes critical to animal movement in the continental United States. *Environ. Sci. Technol.* **57**, 11499–11509 (2023). 37498168 10.1021/acs.est.3c00578 PMC10591311

[64] Land Use & Solar Development, SEIA. Available: https://seia.org/initiatives/land-use-solar-development/.

[65] Sito, E. & Christian, L. *State of the States: Trends and Insights Report*. Available: https://static1.squarespace.com/static/60b7e4e41506593f7f926fe7/t/6643a116b84aa32e721356ad/1715708201864/SoS+Master+Report+FINAL+5.14+1pm+EST.pdf (Wildlands Network, 2024).

[66] Potapov, P. et al. The last frontiers of wilderness: tracking loss of intact forest landscapes from 2000 to 2013. *Sci. Adv.* **3**, e1600821 (2017). 28097216 10.1126/sciadv.1600821 PMC5235335

[67] Theobald, D. M. A general model to quantify ecological integrity for landscape assessments and US application. *Landsc. Ecol.* **28**, 1859–1874 (2013).

[68] Potapov, P. et al. The global 2000-2020 land cover and land use change dataset derived from the Landsat archive: first results. *Front. Remote Sens*. **3**, 10.3389/frsen.2022.856903 (2022).

[69] Dewitz, J. National Land Cover Database (NLCD) 2021. Products: U.S. Geological Survey data release. 10.5066/P9KZCM54 (2021).

[70] Ashraf, U., Morelli, T. L., Smith, A. B. & Hernandez, R. R. Aligning renewable energy expansion with climate-driven range shifts. 10.5061/DRYAD.BNZS7H4J0.

[71] Harvard Law School Environment & Energy Law Program. Trump Rescinded Biden’s Executive Order 14008 Establishing Justice40 Initiative – Environmental and Energy Law Program. Available: https://eelp.law.harvard.edu/tracker/rollback-trump-rescinded-bidens-executive-order-14008-that-established-justice40-initiative/.

[72] Conkling, T. J., Loss, S. R., Diffendorfer, J. E., Duerr, A. E. & Katzner, T. E. Limitations, lack of standardization, and recommended best practices in studies of renewable energy effects on birds and bats. *Conserv. Biol. J. Soc. Conserv. Biol.* **35**, 64–76 (2021). 10.1111/cobi.13457 31913528

[73] Cagle, A. E. et al. Standardized metrics to quantify solar energy-land relationships: a global systematic review. *Front. Sustain*. **3**, 10.3389/frsus.2022.1035705 (2023).

[74] Dunnett, S., Holland, R. A., Taylor, G. & Eigenbrod, F. Predicted wind and solar energy expansion has minimal overlap with multiple conservation priorities across global regions. *Proc. Natl. Acad. Sci. USA* **119**, e2104764119 (2022). 35101973 10.1073/pnas.2104764119 PMC8832964

[75] Seel, J. et al. Utility-scale solar, 2024 edition empirical trends in deployment, technology, cost, performance, PPA pricing, and value in the United States. Available: https://emp.lbl.gov/sites/default/files/2024-10/Utility%20Scale%20Solar%202024%20Edition%20Slides.pdf (Lawrence Berkeley National Laboratory Energy Markets and Policy Department, 2024).

[76] Condon, D. et al. Practitioners’ perceived risks to biodiversity from renewable energy expansion through 2050. *Nat. Human. Soc. Sci. Commun.*10.1057/s41599-025-04558-9 (2025).

[77] Tindale, R. S. & Winget, J. R. Group decision-making. in *Oxford Research Encyclopedia of Psychology*. Available: https://oxfordre.com/psychology/display/10.1093/acrefore/9780190236557.001.0001/acrefore-9780190236557-e-262.

[78] Global Energy Monitor. Global solar power tracker summary data. Available: https://globalenergymonitor.org/projects/global-solar-power-tracker/summary-tables/.

[79] UNEP-WCMC and IUCN. Protected planet: the world database on protected areas (WDPA). Available: http://protectedplanet.net/.

