# Supplementary material for: Practitioner perceptions of biodiversity criteria for solar suitability analyses in the United States
Source: NPJ Biodivers. 2026 May 24;5:24. doi: 10.1038/s44185-026-00133-w (PMC13380617; doi:10.1038/s44185-026-00133-w)
Supplement: Supplementary file 1 — Supplementary information [file 44185_2026_133_MOESM1_ESM.pdf]

Supplementary Information for:

Practitioner perceptions of biodiversity criteria for solar suitability analyses in the United States

Daphne Condon [*,a,b], Michael O. Levin [a,b,c], Adam B. Smith [d], Toni Lyn Morelli [e], Noah Z. Krasner [a,b], Emma Forester [a,b], Chevon Holmes [a,b], Benjamin P. Narwold [a,b], Elizabeth L. Kalies [f], Grace C. Wu [g], Meaghan R. Gade [h], Roland Kays [i,j], Freya Robinson [a,b], Rebecca R. Hernandez [a,b]

[a] Global Ecology and Sustainability Lab, Land, Air & Water Resources Department, University of California, Davis, CA, USA

[b] Wild Energy Center, University of California, Davis, CA, USA

[c] Department of Ecology, Evolution, and Environmental Biology, Columbia University, New York, New York 10027, USA

[d] Center for Conservation and Sustainable Development, Missouri Botanical Garden, St. Louis, MO, USA, ORCID: 0000-0002-6420-1659

[e] U.S. Geological Survey, Northeast Climate Adaptation Science Center, Amherst, MA, USA

[f] The Nature Conservancy, 320 Blackwell Street, Suite 200, Durham, NC, USA

[g] Environmental Studies, University of California Santa Barbara, Santa Barbara, CA, USA

[h] Association of Fish and Wildlife Agencies, Washington DC 20002

[i] North Carolina Museum of Natural Sciences, Raleigh, NC, USA

[j] Department of Forestry and Environmental Resources, NC State University, Raleigh, NC, USA

*Corresponding author

Email: dcondon@ucdavis.edu

Any use of trade, firm, or product names is for descriptive purposes only and does not imply endorsement by the U.S. Government.

Supplementary Item 1: BRC Definitions

Criterion	Definition
National Parks	A type of legally protected area. Congressionally designated protected areas with legal protections against development within their borders.
State Parks	A type of legally protected area. State designated protected areas with legal protections against development within their borders.
National Monuments	A type of legally protected area. Executively or Congressionally designated protected areas with legal protections against development within their borders.
Wilderness Areas	A type of legally protected area. Congressionally designated protected areas with legal protections against development within their borders. Land must be managed to uphold wilderness characteristics.
Inventoried Roadless Areas	A type of legally protected area. United States Forest Service lands that have been identified by government reviews as lands without existing roads that could be suitable for roadless area conservation as wilderness or other non-standard protections. Multiple use activities are still allowed in these areas, but the development of roads within them is limited.
National Wildlife Refuges	A type of legally protected area. Overseen by the US Fish and Wildlife Service, this is the system of public lands and waters set aside to conserve America's fish, wildlife, and plants.
Conservation Easements	A type of legally protected area. A legal agreement between landowners and land trusts or government agencies that permanently limits uses of the land in order to protect its conservation values.
Threatened and Endangered Critical Habitat	This is a legal designation of protected habitat areas for such species defined by the Endangered Species Act.
Proximity to Cliffs	Distance to steep rock faces. Unique landscapes features such as these support unique biota, and thus their presence is relevant when siting solar infrastructure.
Proximity to Springs	Distance to locations where water wells up from an underground source. Unique landscapes features such as these support unique biota, and thus their presence is relevant when siting solar

	infrastructure.
Proximity to Riparian Areas	Distance to the interface between land and rivers or streams. Unique landscapes features such as these support unique biota, and thus their presence is relevant when siting solar infrastructure.
Proximity to Nesting Locations	Depending on the species, some nesting locations have formal protections while others do not. Wildlife use and aggregation areas such as these are vital for the persistence of various taxa in a landscape, and thus their presence is relevant when siting solar infrastructure.
Proximity to Important Bird Areas (IBA's)	IBA's, designated by BirdLife International, are distinct areas that provide essential habitat for one or more species of birds in breeding, wintering, or migration. Wildlife use and aggregation areas such as these are vital for the persistence of various taxa in a landscape, and thus their presence is relevant when siting solar infrastructure.
Proximity to Hibernacula	Distance to places where animals seek refuge. Wildlife use and aggregation areas such as these are vital for the persistence of various taxa in a landscape, and thus their presence is relevant when siting solar infrastructure.
Size of Facility Footprint Relative to Limited Habitat Types	The area of a solar facility’s footprint compared to the area of habitat types in the region the facility is likely to be located. This consideration is focused primarily on the extent to which solar development may displace rarer habitat types.
Habitat Diversity within and around Facility Footprint	Diversity of habitat classifications for the area within and around the footprint of a solar facility.
Habitat Type within and around Facility Footprint	Habitat classification for the area within and around the footprint of a solar facility.
Presence of Species of Greatest Conservation Need	Whether individuals of taxa identified by state wildlife agencies through State Wildlife Action Plans as those in need of most urgent protection can be found within a study area.
Surface Hydrology	The study of surface water movement and the distribution of surface water in space and time.
Subsurface Hydrology	The study of belowground water movement and the distribution of surface water in space and time.
Presence of Taxa of Interest	Whether individuals of taxa of interest can be found within a

	study area. These taxa are identified by practitioners as relevant for biodiversity assessment.
Abundance of Taxa of Interest	How many individuals of taxa of interest can be found within a study area. These taxa are identified by practitioners as relevant for biodiversity assessment.
Distribution of Taxa of Interest	The geographical distribution of taxa of interest across a study area. These taxa are identified by practitioners as relevant for biodiversity assessment.
Range of Taxa of Interest	The area where taxa of interest are typically found during their lifetimes. These taxa are identified by practitioners as relevant for biodiversity assessment.
Diversity of Taxa of Interest	The number of different species that are represented across a study area. These taxa are identified by practitioners as relevant for biodiversity assessment.
Functional Diversity of Taxa of Interest	The value and range of functional traits within a study area. These taxa are identified by practitioners as relevant for biodiversity assessment.
Complementarity/Irreplaceability of Taxa of Interest	The extent to which taxa of interest in a study area utilize different niches, and the distinctness of the roles of taxa of interest within a study area--the fewer overlaps there are in ecological niches/roles played by the species, the higher the complementarity and irreplaceability. These taxa are identified by practitioners as relevant for biodiversity assessment.
Areas of Critical Environmental Concern	A Bureau of Land Management designation that highlights areas where special management attention is needed to protect important historical, cultural, and scenic values, or fish and wildlife or other natural resources.
Habitat Conservation Plan Areas	Habitat Conservation Plans are planning documents designed to accommodate economic development to the extent possible by authorizing the limited and unintentional take of species listed on the Endangered Species Act when it occurs incidental to otherwise lawful activities.
Habitat Management Areas for Threatened and Endangered Species	Areas managed mainly for conservation through management intervention designed to ensure the maintenance of habitats and to meet the requirements of specific species.
Species Identified in State Wildlife Action Plans	Whether individuals of taxa identified in State Wildlife Action Plans can be found within a study area.

Proximity to Forest	Distance to forest land-cover. Forests are used in this case as a proxy for landscapes with potentially high value to biodiversity.
Proximity to Wetlands	Distance to wetland land-cover. Wetlands are used in this case as a proxy for landscapes with potentially high value to biodiversity.
Proximity to Tallgrass Prairie	Distance to Tallgrass Prairie land-cover. Prairies are used in this case as a proxy for landscapes with potentially high value to biodiversity.
Land Use History	Locations that have previously been altered by humans may have less value for wildlife than those previously unaltered by humans. As a result, land use history is relevant when siting solar infrastructure.
Landscape Intactness	A quantifiable estimate of naturalness measured on a gradient of anthropogenic influence.
Landscape Fragmentation	The breaking up of larger areas of natural land cover into smaller, more isolated patches, independent of a change in the total area of natural land cover.
Proximity to Features that may Generate Barrier Effects	Barrier effects involve the impacts of linear infrastructure on the movement of terrestrial animals. This includes roads, fences, and other pieces of linear infrastructure. Solar energy development often requires the construction of road, fence, and transmission linear infrastructure.
Microclimate Diversity	The variety of microclimates present in a study area. The development and operation of a solar facility may alter the diversity of microclimates in a study area.
Landscape Connectivity	The degree to which the landscape facilitates or impedes movement among resource patches. The presence of solar facilities in a landscape may alter that landscape's connectivity.
Migratory Routes	The geographic route along which migratory animals may travel. The presence of a solar facility and removal of habitat within the facility could alter migratory movement for taxa of interest.
Flyways	Flight paths used by large numbers of birds while migrating between their breeding grounds and their overwintering quarters. It is possible that solar infrastructure may have some effect on avian species, as they may appear similar to a large body of water. In addition, construction in flyway stopover points could alter migratory pathways.

Local Movement Routes	Fine-scale movement data for taxa of interest in the study area. The presence of a solar facility and removal of habitat within the facility could alter movement for taxa of interest.
Projections of Future Range Maps for Taxa of Interest	The potential future area where taxa of interest may be found during their lifetimes. While solar infrastructure may not have a large impact on the ranges of taxa of interest in the present, that may change in the future. These taxa are identified by practitioners as relevant for biodiversity assessment.
Projections of Future Climate-Driven Land-Cover and Land-Use Changes	Potential changes to the land-use and land-cover of a landscape due to warming climates. While solar infrastructure may not have a large impact on certain land uses or land covers in the present, that may change in the future.
Soil Properties	Properties of soil include color, texture, structure, porosity, density, consistency, aggregate stability, and temperature.
Climate Resilience	The capacity of a landscape to be resilient in the face of a changing climate, typically measured in the availability of locations more likely to sustain native plants, animals, and natural processes into the future. Solar development could potentially alter a landscape and, in doing so, change its climate resilience.
Ecosystem Services	The capacity of a particular area to provide a specific bundle of ecosystem goods and services within a given time period. Solar development could potentially alter a landscape and, in doing so, change its capacity to supply certain ecosystem services. Ecological functions and benefits are considered in the context of land-use planning (e.g., habitat provision, water regulation), as broadly defined rather than tied to a single classification framework.
Proximity to Vegetation	Distance to any vegetation. Vegetated areas are used in this case as a proxy for landscapes with potentially high value to biodiversity.

Supplementary Item 2: Inventory of biodiversity-relevant criteria used in solar suitability analysis-related studies from the United States as of 2023

BRC Examined	Round Decided	Data Source	Studies
**National Parks**	1	Protected Areas Database of the US (PADUS)	Ref. 1, Ref. 3, Ref. 4, Ref. 7, Ref. 9, Ref. 10, Ref. 13
		State-specific database	Ref. 5
		Solar Programmatic Environmental Impact Statement (EIS) Center	Ref. 11
**State Parks**	1	PADUS	Ref. 1, Ref. 3, Ref. 4, Ref. 7, Ref. 9, Ref. 10, Ref. 13
		State-specific database	Ref. 5
**National Monuments**	1	PADUS	Ref. 1, Ref. 3, Ref. 4, Ref. 7, Ref. 9, Ref. 10, Ref. 13
		National Historic Register	Ref. 5
		Solar Programmatic EIS Center	Ref. 11
**Wilderness Areas**	1	PADUS	Ref. 1, Ref. 3, Ref. 4, Ref. 7, Ref. 9, Ref. 10, Ref. 13
		US Bureau of Land Management (BLM) Western Solar Plan	Ref. 5
		Solar Programmatic EIS Center	Ref. 11
**National Wildlife Refuges**	1	PADUS	Ref. 1, Ref. 3, Ref. 4, Ref. 7, Ref. 9, Ref. 10, Ref. 13
		Solar Programmatic EIS Center	Ref. 11
**Conservation Easements**	1	PADUS	Ref. 1, Ref. 3, Ref. 4, Ref. 7, Ref. 9, Ref. 10, Ref. 13
		National Conservation Easement database	Ref. 13
**Threatened and Endangered Critical Habitat**	1	US Fish and Wildlife Service (FWS) critical habitat database	Ref. 4, Ref. 7, Ref. 11
		FWS Environmental Conservation Online System	Ref. 9

		West-Wide Wind Mapping Project (WWWMP)	Ref. 13
**Areas of Critical Environmental Concern**	1	PADUS	Ref. 1, Ref. 3, Ref. 4, Ref. 7, Ref. 9, Ref. 10, Ref. 13
		Solar Programmatic Environmental Impact Statement (PEIS)	Ref. 11
		WWWMP	Ref. 13
**Habitat Conservation Plan Areas**	1	PADUS	Ref. 1, Ref. 3, Ref. 4, Ref. 7, Ref. 9, Ref. 10, Ref. 13
		Solar Programmatic EIS Center	Ref. 11
**Habitat Management Areas for Threatened and Endangered Species**	1	State/region-specific database	Ref. 4
		FWS critical habitat database	Ref. 9, Ref. 13
		Solar Programmatic EIS Center	Ref. 11
		WWWMP	Ref. 13
**Proximity to Important Bird Areas (IBAs)**	2	Audubon Important Bird Areas	Ref. 4, Ref. 9, Ref. 13
**Presence of Species of Greatest Conservation Need**	2	State/region-specific database	Ref. 4, Ref. 13
**Presence of Taxa of Interest**	2	NatureServe	Ref. 4, Ref. 13
		Solar Programmatic EIS Center	Ref. 11
		State/region-specific database	Ref. 13
**Landscape Intactness**	2	Theobald human modification index (HMI)	Ref. 1
		The Disappearing West database	Ref. 4
		The Nature Conservancy (TNC) Resilient & Connected Network	Ref. 13

**Landscape Connectivity**	3	WWWMP	Ref. 4,
		BLM Western Solar Plan	Ref. 5,
		Solar Programmatic EIS Center	Ref. 11
		TNC Resilient & Connected Network	Ref. 13
**Migratory Routes**	3	State/region-specific database	Ref. 13

All consensus core criteria come from rounds 1 and 2. References pertain to the following studies: Ref. 1: Patankar et al. [37]; Ref. 2: Kwak et al. [20]; Ref. 3: Katkar et al. [52]; Ref. 4: Wu et al. [34]; Ref. 5: Majumdar and Pasqualetti [54]; Ref. 6: Hoffacker et al. [74]; Ref. 7: Hernandez et al. [38]; Ref. 8: Brewer et al. [35]; Ref. 9: Wu et al. [53]; Ref. 10: Tisza et al. [75]; Ref. 11: Stoms et al. [76]; Ref. 12: Hott et al. [36]; Ref 13: Wu et. al. [33].

Supplementary Item 3: Summaries of examined US-based solar suitability analysis-related studies

Solar Suitability Analysis	Study Description
Ref. 1: Land use trade-offs in decarbonization of electricity generation in the American West.	This paper assessed the quality, cost-effectiveness, and land-use conflict risks of wind and solar energy sites within the Western Electricity Coordinating Council. The findings revealed that less than 4% of suitable solar sites and less than 17% of wind sites are highly energy-productive and cost-effective, while less than 53% of solar sites and less than 85% of wind sites present development risks. Although avoiding land-use conflict-prone areas could increase system costs and complicate strategic siting, state collaboration—particularly among Arizona, California, Nevada, and Utah for solar, and Arizona, Colorado, Oregon, and Wyoming for wind—could help mitigate land-use conflicts during the clean energy transition
Ref. 2: A large scale multi criteria suitability analysis for identifying solar development potential: A decision support approach for the state of Illinois, USA	This study developed a decision-support tool to identify suitable public lands for solar energy development in Illinois. Using geospatial technologies and suitability analysis, the paper evaluated environmental, socioeconomic, and energy productivity factors to support state agency-led solar expansion. The findings emphasize the importance of high-resolution data for large-scale comparisons and site-specific feasibility analysis. Additionally, the study highlights the value of a Planning Support System in facilitating decision-making and enhancing stakeholder engagement
Ref. 3: Strategic land use analysis for solar energy development in New York State	This paper addressed the challenge of balancing utility-scale solar energy development with the preservation of agricultural lands in New York, examining suitability using geospatial information systems and multi-criteria analysis techniques. The authors estimate that 44% of utility-scale solar energy development is sited on agricultural land, while 84% of undeveloped land suitable for utility-scale solar energy is agricultural. The authors suggested protecting prime farmland, implementing agrivoltaics, and incentivizing the use of marginal lands to mitigate land use conflicts. It also emphasized the need for community involvement in decision-making to guide effective solar expansion in New York.

Ref. 4: Low-impact land use pathways to deep decarbonization of electricity	This study examined land-use trade-offs in meeting California's clean energy goals. Using ecological and agricultural data from 11 western US states, it modeled environmental constraints on wind, solar, and geothermal energy generation. The findings showed that California can achieve its energy targets; however, development costs, technology choices, and deployment layout depend on land protections and out-of-state resources. Neglecting land availability in energy planning could result in higher economic and environmental costs, and increased uncertainty in achieving climate goals
Ref. 5: Analysis of land availability for utility-scale power plants and assessment of solar photovoltaic development in the state of Arizona, USA	This paper investigated suitable land for utility-scale photovoltaic solar development in Arizona using geographical information systems and multiple-criteria analysis to address challenges in meeting the growing demand for clean energy. The study found that less than 2% of Arizona's land is "excellent" for solar, primarily on private or state trust lands. Urbanization poses a major challenge, depleting suitable land and requiring alternative solutions. The researchers posit that Arizona could become a regional energy hub if these lands are developed.
Ref. 6: Land-Sparing Opportunities for Solar Energy Development in Agricultural Landscapes: A Case Study of the Great Central Valley, CA, United States	This study explored the potential of deploying solar energy on nonconventional lands to minimize land-use conflicts with agriculture and conservation in the Central Valley, California. Researchers identified 8,415 km^2 of land, including built environments, salt-affected areas, contaminated land, and water reservoirs, suitable for solar development. Harnessing these areas could generate enough energy to surpass California's projected 2025 electricity demands up to 13x, demonstrating a viable strategy for balancing renewable energy goals with other land-use needs.
Ref. 7: Solar energy development impacts on land cover change and protected areas	This study examined the land-use impacts of utility-scale solar energy installations in California, focusing on their siting concerning natural environments, agricultural lands, and protected areas. The research revealed that most utility-scale solar installations are primarily located in shrublands and scrublands, followed by agricultural areas. The authors note that less than 15% of installations are sited in "compatible" areas. The findings highlighted the need for improved integration of energy, food, and conservation goals in solar energy siting decisions to achieve

	environmental and energy compatibility and harness potential co-benefit opportunities.
Ref. 8: Using GIS analytics and social preference data to evaluate utility-scale solar power site suitability	This paper presented a geographical information system-based multiple-criteria analysis for identifying optimal locations for utility-scale solar energy projects in the southwestern United States. The assessment combined proximity raster layers, terrain datasets, and solar irradiance information to create high-resolution maps displaying solar energy potential from "poor" to "excellent" across selected counties. By integrating social preference data collected from surveys, the research demonstrated that considering public acceptance significantly reduces the suitable area for solar development, highlighting the importance of stakeholder engagement in utility-scale solar energy project siting.
Ref. 9: Incorporating land-use requirements and environmental constraints in low-carbon electricity planning for California	This paper assessed the land-use implications of 2050 energy decarbonization scenarios for California. Using a spatially explicit model, the study revealed that, while California has sufficient land for solar and geothermal targets, high-quality wind and solar lands often overlap with high conservation value areas. The research concluded that basing siting decisions on environmentally constrained, long-term renewable energy development goals can lead to better conservation outcomes and improved land-use efficiency compared to the existing planning approach.
Ref. 10: GIS based analysis for photovoltaic deployment in the Southeast US	This study assessed the technical potential for utility-scale photovoltaic solar deployment in four southeastern U.S. states, comparing results with a prior study. Key findings include: (1) significant land is available for utility-scale solar in all four states, and (2) technical and electricity generation potential (GWh) were calculated for each state using 15% panel efficiency. The research provides a preliminary characterization of suitable areas for PV deployment, recommending further techno-economic investigation at smaller scales with additional criteria like aspect.

Ref. 11: Siting solar energy development to minimize biological impacts	This paper presented a spatial multi-criteria analysis method for modeling conflict risk between solar energy development and biodiversity conservation in California. The study identified compatible sites for solar energy as those that are of low conservation value and require minimal off-site impacts for transmission infrastructure connection. Results indicated sufficient compatible land exists in flat, non-urban areas of Californian deserts to meet the 2040 solar energy target of 8.7 gigawatts of installed capacity.
Ref. 12: GIS-based spatial analysis for large-scale solar power and transmission line issues: Case study of Wyoming, U.S.	This study presented a model combining geographical information systems and multi-criteria analysis to identify suitable locations for large-scale solar energy projects in Wyoming. The research overlaid solar resource data, population areas, and transmission line data with suitable location files, revealing that many suitable areas are close to cities and existing transmission lines. The study concluded that Wyoming has ample solar resources and transmission capacity to support solar energy projects for commercial and residential sectors.
Ref. 13: Minimizing habitat conflicts in meeting net-zero energy targets in the western United States	This study modeled high-resolution energy and land-use data to assess the implications of land and ocean use in achieving net-zero greenhouse gas emissions in the western US by 2050. The findings revealed that a "High Electrification" scenario most efficiently uses electricity generation and requires the least land and ocean area. Importantly, the research showed that implementing stronger land- and ocean-use protections to meet net-zero targets increases energy system costs by only 3%, while significantly reducing conflicts and habitat loss in high conservation value areas

Supplementary Item 4: Example Jamboard slides from the Delphi prioritization activity

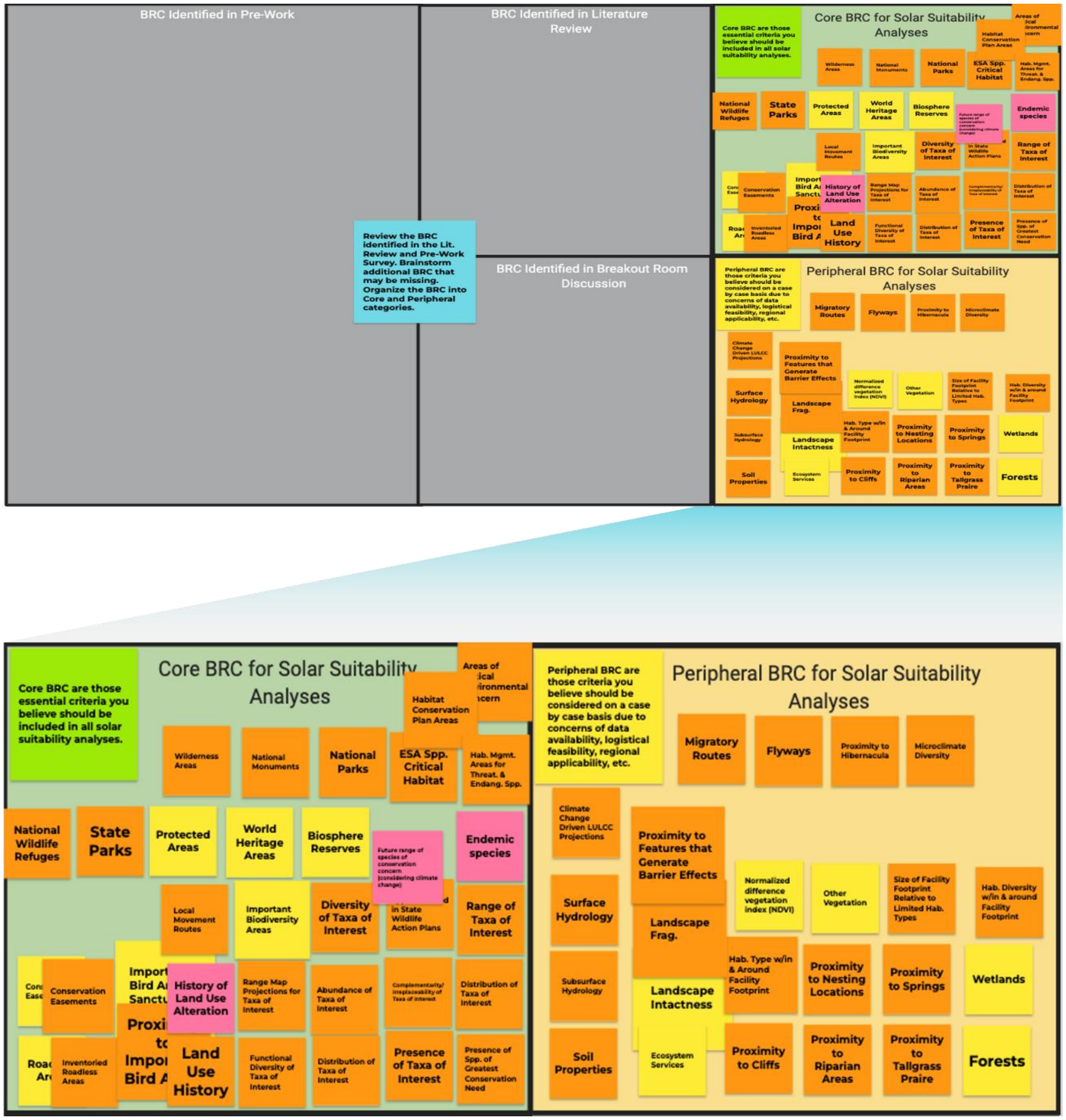

Figure a | Example breakout room Jamboard panel, Delphi round 1. Practitioners were asked to move the pre-identified literature review criteria (yellow sticky notes) and pre-survey criteria (orange sticky notes) to the "core" criteria box (green, top right) or "peripheral" criteria box (yellow, bottom right). Pink sticky notes represented practitioner-identified biodiversity-relevant criteria. Core and peripheral BRC boxes are enlarged to enhance readability.

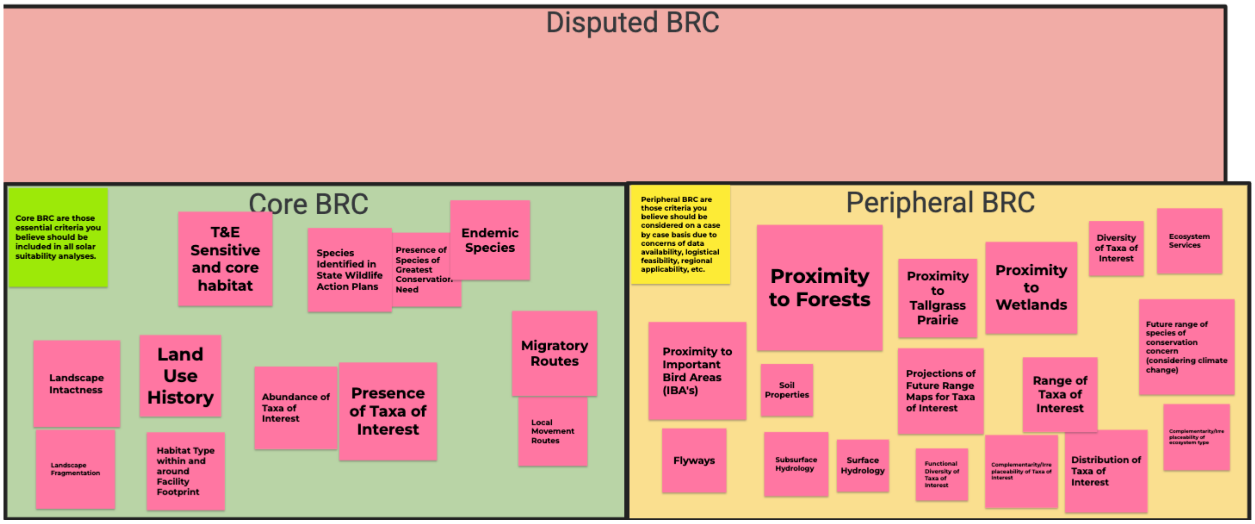

Figure b | Example breakout room Jamboard panel, Delphi round 2. Practitioners were asked to move the disputed biodiversity-relevant criteria to the "core" criteria box (green, bottom left) or "peripheral" criteria box (yellow, bottom right).
